# Bone Development in Transgender Adolescents Treated With GnRH Analogues and Subsequent Gender-Affirming Hormones

**DOI:** 10.1210/clinem/dgaa604

**Published:** 2020-09-10

**Authors:** Sebastian E E Schagen, Femke M Wouters, Peggy T Cohen-Kettenis, Louis J Gooren, Sabine E Hannema

**Affiliations:** 1 Department of Pediatric Endocrinology, VU University Medical Center, 1081 HV Amsterdam, & Leiden University Medical Center, ZA Leiden, the Netherlands; 2 Department of Pediatric Endocrinology, VU University Medical Center, HV Amsterdam, the Netherlands; 3 Department of Medical Psychology, Amsterdam UMC, VU University, HV Amsterdam, the Netherlands; 4 Department of Endocrinology, VU University Medical Center, HV Amsterdam, the Netherlands; 5 Department of Pediatrics, Willem-Alexander Children Hospital, Leiden University Medical Center, ZA Leiden, the Netherlands; 6 Department of Pediatric Endocrinology, Sophia Children’s Hospital, Erasmus Medical Centre, CN Rotterdam, Netherlands

**Keywords:** bone mineral density, bone, GnRH analogue, sex steroids, gender dysphoria, transgender, adolescents

## Abstract

**Context:**

Hormonal interventions in adolescents with gender dysphoria may have adverse effects, such as reduced bone mineral accrual.

**Objective:**

To describe bone mass development in adolescents with gender dysphoria treated with gonadotropin-releasing hormone analogues (GnRHa), subsequently combined with gender-affirming hormones.

**Design:**

Observational prospective study.

**Subjects:**

51 transgirls and 70 transboys receiving GnRHa and 36 transgirls and 42 transboys receiving GnRHa and gender-affirming hormones, subdivided into early- and late-pubertal groups.

**Main Outcome Measures:**

Bone mineral apparent density (BMAD), age- and sex-specific BMAD z-scores, and serum bone markers.

**Results:**

At the start of GnRHa treatment, mean areal bone mineral density (aBMD) and BMAD values were within the normal range in all groups. In transgirls, the mean z-scores were well below the population mean. During 2 years of GnRHa treatment, BMAD stabilized or showed a small decrease, whereas z-scores decreased in all groups. During 3 years of combined administration of GnRHa and gender-affirming hormones, a significant increase of BMAD was found. Z-scores normalized in transboys but remained below zero in transgirls. In transgirls and early pubertal transboys, all bone markers decreased during GnRHa treatment.

**Conclusions:**

BMAD z-scores decreased during GnRHa treatment and increased during gender-affirming hormone treatment. Transboys had normal z-scores at baseline and at the end of the study. However, transgirls had relatively low z-scores, both at baseline and after 3 years of estrogen treatment. It is currently unclear whether this results in adverse outcomes, such as increased fracture risk, in transgirls as they grow older.

Over the last decades, children diagnosed with gender dysphoria have increasingly come to the attention of the psychomedical care system and clinicians recognize their suffering, aggravated by the somatic changes of puberty (1, 2). The development of secondary sex characteristics can be temporarily halted with gonadotropin-releasing hormone analogue (GnRHa) treatment (3). This offers the adolescent the opportunity to explore their wish to pursue gender-affirming treatment, while no longer experiencing the agonizing development of secondary sex characteristics due to endogenous puberty, which are incongruent with gender identity. Birth-assigned girls must be at least in Tanner breast stage 2 with clear palpable mammary tissue, while birth-assigned boys must have reached Tanner stage G2 before initiating treatment with GnRHa (3, 4). If no contraindications exist, sex steroids consistent with the affirmed gender are added to the GnRHa treatment at an age where adolescents can give informed consent to such treatment, usually at approximately 16 years (3). There is much discussion about this age, since 16 years is considered a late age to induce puberty in adolescents.

In young adults, peak bone mass (PBM) is higher in men than in women (5). Sex steroids play an essential role in the establishment of gender differences in bone mass, both through direct effects and indirect effects, for example, via differences in muscle mass and insulin-like growth factor (6). Puberty is an important period in determining adult bone mineral content (6). Together, these findings strengthen the notion that maximizing bone mineral accrual during adolescence may be important in the prevention of osteoporosis and fractures at older age.

One of the primary concerns when using GnRHa in adolescents for a prolonged period of time is the potential decrease in bone mineral density (BMD) (3, 7). The suppression of the endogenous sex steroids to stop pubertal development, as recommended by current guidelines, may potentially interfere with the normal pubertal bone mass increment and reduce PBM. Therefore, assessment of BMD every 1 to 2 years is recommended (3). Three studies in adolescents diagnosed with gender dysphoria receiving GnRHa and gender-affirming hormone treatment reported decreases in areal BMD (aBMD) and bone mineral apparent density (BMAD) z-scores during GnRHa treatment, although not all significant (8-10). Little difference was noted in change of BMAD z-scores between early- and late-pubertal groups as defined by bone age (8). Catch-up of bone mineral accrual during subsequent gender-affirming hormone treatment may be incomplete (8-10). One study investigated bone markers and showed a decrease of carboxyterminal cross-linked telopeptide of type I collagen (1CTP) and N-terminal propeptide of type-1 collagen (P1NP) during GnRHa and during subsequent gender-affirming hormone treatment which was interpreted as evidence of decreased bone turnover (8). All these studies compared data at the start of GnRHa treatment, at the start of gender-affirming hormones and one endpoint, either 12–24 months after the start of gender-affirming hormone therapy or age 22 years. However, this does not provide information on the course of BMD during treatment. Do BMD z-scores continue to decline with prolonged use of GnRHa? How long do BMD z-scores continue to increase during GAH treatment? These questions remain unanswered. Now that increasing numbers of adolescents undergo this treatment, possibly starting at younger ages, there is a clear need for such data. Therefore we set out to describe the course of BMD during 2 years of GnRHa therapy and during 3 years of subsequent gender-affirming hormone treatment in a large group of adolescents diagnosed with gender dysphoria, with measurements at yearly intervals. We also investigated whether the outcome was influenced by the pubertal stage, as defined by Tanner stage, at which GnRHa treatment was started. In addition, we report data from a small subgroup with more prolonged GnRHa treatment.

## Methods

### Subjects and protocol

Subjects were adolescents fulfilling *Diagnostic and Statistical Manual of Mental Disorders, Fourth Edition, Text Revision* (*DSM-IV-TR*) criteria for gender identity disorder (the term used at the time) (11) and eligible for treatment according to existing guidelines at that time (4, 12, 13). The design of the study was observational and prospective, and individuals were included from 1998 to 2009. The first phase of treatment consisted of intramuscular injections of GnRHa 3.75 mg (Triptorelin-CR (Ferring Pharmaceuticals, Denmark). The first 2 injections were administered with a 2-week interval followed by injections every 4 weeks to suppress endogenous sex steroid production. To induce female pubertal development in transgirls, oral estrogens were prescribed in an increasing dosage over a period of 2 years as previously described (4). Male puberty in transboys was induced by administering Sustanon (a mixture of testosterone propionate, -fenylpropionate, -isocaproate and -decanoate) intramuscularly in increasing doses over a period of 2 years (14). In subjects who were 16 years of age or older at the start of pubertal suppression, gender-affirming hormones were started at half the adult dose and increased to the adult dose after 6 months. A dose of 2 mg 17beta-estradiol per day and 125 mg testosterone-esters per 2 weeks was considered an adult dose. From 45 subjects, some data were also included in previous studies by Vlot et al (8) and Klink et al (10), but those studies only reported results at 3 time points: at the start of GnRHa, at the start of gender-affirming hormones, and after 2 years of gender-affirming hormones (8) or age 22 years (10), and they did not describe a detailed course of BMD and bone markers over several years of GnRHa or gender-affirming hormone treatment.

Different effects of treatment might be expected depending on the pubertal stage at baseline. A previous study used a bone age cutoff of 14 and 15 years for transboys and transgirls, respectively, to define early- and late-pubertal groups (8). However, especially for transboys, bone age 14 years signifies the final stages of puberty and near completion of linear growth rather than midpuberty. In the current study, Tanner stage was used to define early- and late-pubertal groups, with the early-pubertal group defined as Tanner stage 2 or 3 at the start of GnRHa treatment, and the late-pubertal group as Tanner stage 4 or 5.

### Bone densitometry

Dual-energy x-ray absorptiometry (DXA) was performed before GnRHa administration and then every subsequent year using Hologic QDR 4500 (Holologic Inc., Waltham, MA, USA). Likewise, at the start of gender-affirming hormone treatment, a DXA scan was performed, with yearly measurements thereafter. Areal BMD (aBMD, g/cm^2^) of the lumbar spine, nondominant hip, and whole body, as well as the bone mineral content of the whole body (BMC-WB, g) were measured. To calculate z-scores based on age and sex, the National Health and Nutrition Examination Surveys (NHANES) references values were used. Because changes in aBMD might partly be due to altered growth during treatment, we also studied BMAD (g/cm^3^) calculated as described by Ward et al (15). BMAD z-scores were calculated using LMS data from an English reference population (15). To calculate z-scores the reference population of the birth-assigned sex was used. For adolescents older than 17 years no reference values of BMAD are available; therefore, reference values of 17 year-olds were used to calculate the z-score at older ages (15).

### Serum bone markers

Markers of bone formation (P1NP, P3NP, and osteocalcin) and of bone resorption (1CTP) were determined in fasting blood samples, drawn before noon on the same days as the DXA scans, and stored at −20 °C.

Osteocalcin was measured by an immunometric assay (Colorimetric, BioSource, Nivelles, Belgium) (lower detection limit of 0.4 nmol/L; inter-assay coefficient of variation (CV) for the whole range <10%). Serum 1CTP, P1NP, and P3NP levels were measured using a radioimmunoassay (Orion Diagnostica, Espoo, Finland). The lower ranges of detection were 1 µg/L for 1CTP, 5 µg/L for P1NP, and 1µg/L for P3NP. The inter-assay CV for the whole range of 1CTP was 7% and for P1NP 8%. The CV for P3NP was 6% at 4.2 µg/L and 8% at 6.2 µg/L.

### Statistical analyses

Independent *t* tests were used to ascertain differences between the ages of the transgirls and transboys. To analyze changes in BMAD over time, data were analyzed using a linear mixed model. A full factorial model was chosen as fixed part of the model, ie, a model consisting of time (3 or 4 levels), pubertal stage (early/late), and sex and all possible interactions (ie, three 2-way and one 3-way interactions). An unstructured covariance matrix was used as random part of the model. An advantage of the linear mixed model approach above traditional repeated measurements analysis of variance (ANOVA) is that all acquired data are included in the analyses and no data are lost due to incomplete data sets.

Differences in aBMD during a more prolonged period of GnRHa treatment were calculated using the related samples Wilcoxon Signed Ranked test.

All data on BMAD, and z-scores are presented as estimated marginal means and standard error of the mean. The statistical package was SPSS 22.0 (SPSS Inc., Chicago, IL, USA).

### Ethical approval

The study was placed on the International Standard Randomized Controlled Trial Number register and ascribed registration number ISRCTN 81574253 (www.isrctn.com). Approval by the local medical ethical committee was obtained. Informed consent for the study was obtained from all adolescents, and if aged <18 years also from their parents.

## Results

A total of 54 transgirls and 73 transboys started treatment according to this protocol. For 51 transgirls and 70 transboys, DXA scans were available at the start of GnRHa administration and these individuals were included in the analyses. There were no significant differences between the ages of the transgirls and the transboys at the start of GnRHa administration (Table 1).

**Table 1.** Characteristics at the Start of GnRHa Treatment and at the Start of Gender-Affirming Hormone Treatment

Start GnRHa		Transgirls (n = 51)	Transboys (n = 70)	*P* value
	Age in years, mean ± SD	14.1 ± 1.7	14.5 ± 2.0	n.s.
	Pubertal group: Early/late	15/36	14/56	n.s.
	Height in cm, mean ± SD	169.0 ± 8.9	162.2 ± 8.8	<0.001
	Weight in kg, mean ± SD	57.9 ± 12.9	56.2 ± 14.7	n.s.
	BMI in kg/m^2^, mean ± SD	20.1 ± 3.3	21.3 ± 4.2	n.s.
	Serum estradiol in pmol/L, median [IQR]		Early: 113.5 [63.5–129.3]	
			Late: 121 [83.5–231.5]	
	Serum testosterone in nmol/L, median [IQR]	Early: 3.8 [2.15–6.15] Late: 13 [10.3–17.8]		
**Start gender-affirming hormones**		Transgirls (n = 36)	Transboys (n = 42)	
	Age in years, mean ± SD	16.2 ± 1.2	16.9 ± 1.1	0.005
	Pubertal group: Early/late	10/26	5/37	n.s.
	Duration of GnRHa use before start GAH, years	2.0 ± (0.94)	1.8 ± (1.11)	n.s.
	Height in cm, mean ± SD	176.5 ± 7.3	167.1 ± 7.4	0.005
	Weight in kg, mean ± SD	66.7 ± 11.9	63.5 ± 11.5	n.s.
	BMI in kg/m^2^, mean ± SD	21.1 ± 3.2	22.8 ± 4.0	n.s.

Abbreviations: BMI, body mass index; GAH, gender-affirming hormones; GnRHa, gonadotropin-releasing hormone analogue; IQR, interquartile range; n.s., not significant; SD, standard deviation.

A total of 36 transgirls and 42 transboys received gender-affirming hormone treatment in addition to GnRHa treatment. The transboys were slightly but significantly older at start of gender-affirming hormone treatment than the transgirls (Table 1). The ratio of subjects who were in early and in late puberty was not different in the group evaluated for the effects of gender-affirming hormone treatment compared with the group analyzed during GnRHa treatment alone.

Anthropometric data and data on pubertal development of the subjects at baseline are shown in Table 1. All adolescents had sex characteristics typical of the sex assigned at birth and none had signs of a difference/disorder of sex development. None of the adolescents had a bone fracture during the study.

### Changes during 2 years of GnRHa treatment

#### Bone mineral apparent density.

**Figure 1.**
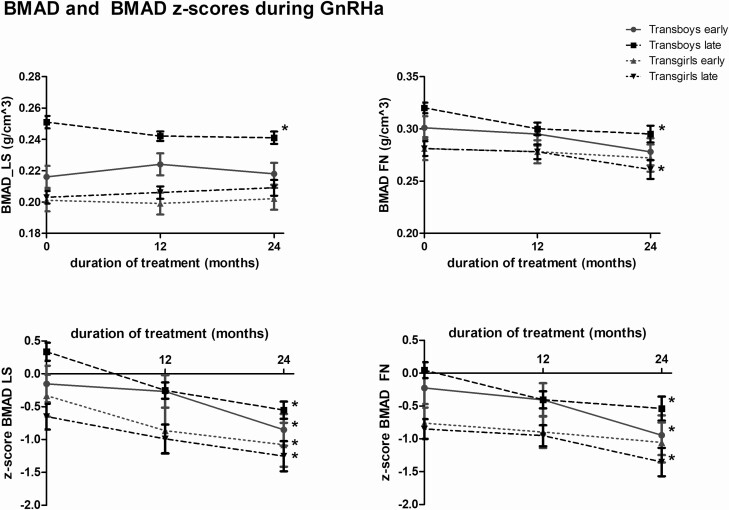
Estimated marginal means and standard error of the mean of BMAD prior to and during 2 years of GnRHa administration in transgirls and transboys. Significant changes during the 2 years of GnRHa administration are indicated by an asterisk. Abbreviations: BMAD: bone mineral apparent density; FM, femoral neck; LS, lumbar spine.

Changes in aBMD and aBMD z-scores are shown in Table 2. BMAD of the lumbar spine did not change during 2 years of GnRHa treatment in the transgirls or the early pubertal transboys (*P* = 0.84*, P* = 0.09, and *P* = 0.69, respectively) (see Fig. 1, Table 2). In the late-pubertal transboys, a small but significant decrease in BMAD of the lumbar spine was found.

**Table 2.** aBMD and BMAD During 2 Years of GnRHa Treatment

Transgirls						
	Early Pubertal		Late-Pubertal			
	0 mo	24 mo	0 mo	24 mo	*p*1	*p*2
aBMD_LS g/cm^2^	0.73 (0.03)	0.75(0.03)	0.79 (0.02)	0.82 (0.02)	<0.05	<0.05
Z-score	−0.67 (0.26)	−1.26 (0.24)	−0.33 (0.17)	−0.92 (0.17)	<0.05	<0.05
aBMD_hip g/cm^2^	0.81 (0.03)	0.86 (0.03)	0.87 (0.02)	0.89 (0.02)	<0.05	n.s.
Z-score	−0.49 (0.24)	−0.93 (0.21)	−0.43 (0.16)	−1.01 (0.15)	<0.05	<0.05
Whole body BMD g/cm^2^	0.90 (0.02)	0.92 (0.02)	0.95 (0.01)	0.95 (0.01)	<0.05	n.s.
Z-score	−0.56 (0.24)	−1.51 (0.20)	−0.51 (0.16)	−1.62 (0.15)	<0.05	<0.05
BMAD_LS g/cm^3^	0.20 (0.01)	0.20 (0.01)	0.20 (0.01)	0.21 (0.0!)	n.s.	n.s.
Z-score	−0.33 (0.33)	−1.19 (0.34)	−0.65 (0.20)	−1.21 (0.22)	<0.05	<0.05
BMAD_hip g/cm^3^	0.28 (0.01)	0.27 (0.01)	0.28 (0.01)	0.26 (0.01)	n.s.	<0.05
Z-score	−0.94 (0.27)	−1.23 (0.35)	−1.01 (0.17)	−1.56 (0.25)	n.s.	<0.05
Transboys						
	Early-pubertal		Late-pubertal			
	0 mo	24 mo	0 mo	24 mo	*p*1	*p*2
aBMD_LS g/cm^2^	0.75 (0.03)	0.80 (0.03)	0.95 (0.01)	0.92 (0.01)	<0.05	<0.05
Z-score	−0.28 (0.27)	−1.04 (0.26)	0.38 (0.14)	−0.71 (0.14)	<0.05	<0.05
aBMD_hip g/cm^2^	0.79 (0.03)	0.83 (0.03)	0.93 (0.01)	0.89 (0.02)	<0.05	<0.05
Z-score	0.09 (0.26)	−0.50 (0.24)	0.46 (0.13)	−0.56 (0.13)	<0.05	<0.05
Whole body BMD g/cm^2^	0.88 (0.02)	0.92 (0.02)	1.03 (0.01)	1.01 (0.01)	<0.05	<0.05
Z-score	−0.28 (0.27)	−0.82 (0.24)	0.66 (0.13)	−0.40 (0.13)	<0.05	<0.05
BMAD_LS g/cm^3^	0.22 (0.01)	0.22 (0.01)	0.25 (0.01)	0.24(0.01)	n.s.	<0.05
Z-score	−0.15 (0.29)	−0.86 (0.30)	0.33 (0.14)	−0.56 (0.17)	<0.05	<0.05
BMAD_hip g/cm^3^	0.30 (0.01)	0.28 (0.01)	0.32 (0.01)	0.30 (0.01)	<0.05	<0.05
Z-score	−0.23 (0.25)	−0.94 (0.30)	0.04 (0.12)	−0.54 (0.18)	<0.05	<0.05

aBMD and BMAD during 2 years of GnRHa treatment. Values are presented as estimated marginal means ± standard error. *p1* represents the *P* value between the start and after 2 years of treatment for the early pubertal groups. *p2* represents the *P* value between start and after 2 years of treatment for the late-pubertal groups.

For changes per year of treatment see Fig. 1.

Abbreviations: aBMD, areal bone mineral density; BMAD, bone mineral apparent density; BMD, bone mineral density; LS, lumbar spine.

BMAD of the femoral neck showed a significant decrease in the late-pubertal transgirls and in both groups of transboys (*P* = 0.007, *P* = 0.015, and *P* < 0.001, respectively) (see Fig. 1, Table 2). The small decrease in the early pubertal transgirls was not significant (*P* = 0.31).

#### Bone mineral apparent density z-scores.

At the start, z-scores of the BMAD at both locations were higher in the transboys than in the transgirls. The BMAD z-score of the lumbar spine significantly decreased in all 4 groups (*P* ≤ 0.001) (see Fig. 1, Table 2). The BMAD z-scores of the femoral neck significantly decreased in all groups (*P* = 0.006, *P* = 0.002, and *P* < 0.001) except for the early-pubertal transgirls (*P* = 0.25). Four transgirls had a z-score of the hip below −2 after 2 years of GnRHa treatment and 3 individuals had a z-score of the lumbar spine below −2. Two transboys had a z-score of the hip below −2 whereas none of the transboys had a z-score of the lumbar spine below −2 after 2 years of GnRHa treatment.

#### Bone mineral density during prolonged GnRHa treatment.

Because the average age at the start of GnRHa treatment was more than 14 years, most individuals were not treated with GnRHa for more than 2 years before gender-affirming hormone treatment was started. However, a few younger individuals were treated for up to 4 years. The aBMD values of the lumbar spine and hip in 4 transboys and 11 transgirls remained stable during 3 years of GnRHa treatment. The z-scores on the other hand declined (Table 3).

**Table 3.** aBMD and aBMD Z-Scores During 3 Years of GnRHa Treatment

Sex	Age at Start (Range)	Duration GnRHa(yrs)		Start	12 Months	24 Months	36 Months	*P*
Transgirls	12.6 (12.1-12.8)	3.45 (0.43)	aBMD LS (g/cm^2) mean(± SD) (n = 4)	0.73 (0.9)	.74 (0.10)	0.77 (0.11)	0.77 (0.11)	0.14
			Z-score LS mean (± SD) (n = 4)	−0.43 (1.41)	−0.92 (1.40)	−1.05 (1.31)	−1.15 (1.00)	0.07
			aBMD Hip (g/ cm^2) mean (± SD) (n = 4)	0.80 (0.04)	0.82 (0.4)	0.83 (0.05)	0.85 (0.06)	0.07
			Z-score hip mean (± SD) (n = 4)	−0.18 (0.50)	−0.65 (0.34)	−1.08 (0.42)	−1.08 (0.42)	0.007
Transboys	12.7 (11.9-14.0)	3.30 (0.50)	aBMD LS (g/cm^2) mean (± SD) (n)	0.85 (0.13) (11)	0.88 (0.10) (11)	0.90 (0.11) (11)	0.90 (0.9) (11)	0.29
			Z-score LS mean (± SD) (n)	0.42 (1.01) (9)	−0.52 (0.83) (10)	−0.35 (0.96) (11)	−0.53 (0.78) (11)	0.008
			aBMD Hip (g/ cm^2) mean (± SD) (n)	0.88 (0.09) (9)	0.88 (0.71) (11)	0.87 (0.08) (11)	0.88 (0.09) (11)	0.95
			Z-score hip mean (± SD) (n)	0.86 (0.71) (8)	0.40 (0.71) (8)	−0.18 (0.67) (9)	−0.30 (0.67) (10)	0.12

Abbreviations: aBMD, areal bone mineral density; LS, lumbar spine; SD, standard deviation.

#### Serum bone markers.

**Figure 2.**
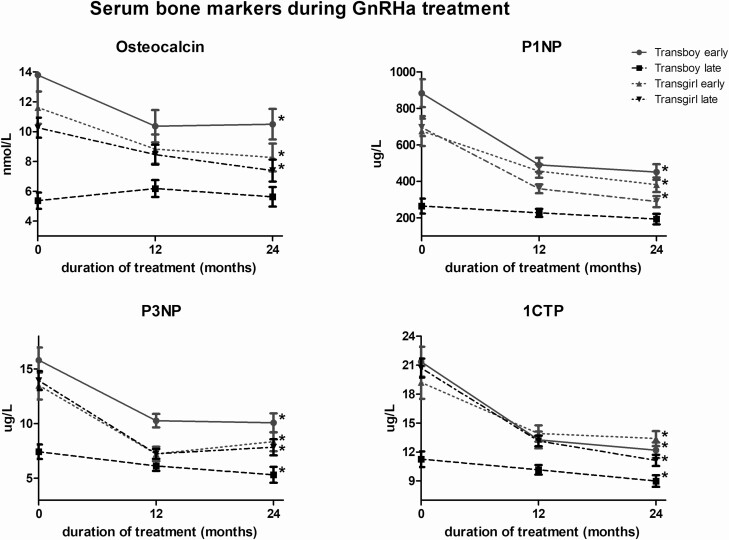
Estimated marginal means and negative standard error of the mean of osteocalcin, P1NP, P3NP, and 1CTP prior to and during 2 years of GnRHa administration in transgirls and transboys. Significant changes during the 2 years of GnRHa administration are indicated by asterisk.

At baseline, there were no significant differences in serum levels of any of the 4 bone markers (P1NP, P3NP, osteocalcin, 1CTP) between the early- and late-pubertal groups of transgirls (Fig. 2). In the transboys, baseline serum levels of all 4 bone markers were significantly higher in those in early puberty compared to those in later puberty.

After 2 years of GnRHa treatment serum levels of all 4 bone markers showed a significant decrease in both groups of transgirls and in early-pubertal transboys, which was most marked during the first year of treatment (Fig. 2). Serum levels of P3NP and 1CTP showed a smaller but significant decrease in late-pubertal transboys whereas serum levels of P1NP and osteocalcin did not change in this group.

### Changes during 3 years of gender-affirming hormone treatment

After an average of 1.89 years (± 1.03 year) of GnRHa administration, gender-affirming hormones were added to the treatment. Both early-pubertal groups were on GnRHa for a significantly longer time (2.5 years in transgirls (n = 7) and 4.0 years in transboys (n = 3)) when compared with both late-pubertal groups (1.5 years in transgirls and 1.7 years in transboys) (*P* < 0.001).

#### Bone mineral apparent density.

**Figure 3.**
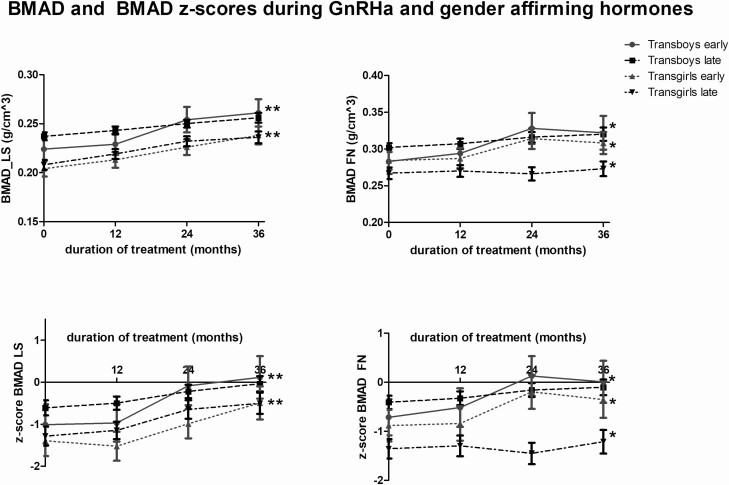
Estimated marginal means and standard error of the mean of BMAD prior to and during 3 years of GnRHa + gender-affirming treatment in transgirls and transboys. Significant changes during the 3 years of GnRHa + gender-affirming treatment are indicated by an asteriks.

Changes in aBMD and aBMD z-scores are shown in Table 4. A significant increase in BMAD of the lumbar spine was found in all 4 groups (*P* < 0.001) after 3 years of gender-affirming hormone treatment (Fig. 3, Table 4). The BMAD of the femoral neck showed a significant increase in both groups of transgirls and in the early-pubertal transboys (*P* < 0.05). In the late-pubertal transboys the increase was not significant.

**Table 4.** aBMD and BMAD During 3 Years of Gender-Affirming Hormone Treatment in Addition to GnRHa Treatment

Transgirls						
	Early-Pubertal		Late-Pubertal			
	0	36	0	36	*p*1	*p*2
aBMD_LS g/cm^2^	0.77 (0.03)	0.95 (0.04)	0.83 (0.02)	0.95 (0.03)	<0.05	<0.05
Z-score	−1.37 (0.30)	−0.82 (0.39)	−0.99 (0.19)	−1.05 (0.25)	<0.05	n.s.
aBMD_hip g/cm^2^	0.87 (0.03)	1.02 (0.04)	0.88 (0.02)	0.96 (0.02)	<0.05	<0.05
Z-score	−0.99 (0.23)	−0.09 (0.28)	−0.86 (0.14)	−0.70 (0.18)	<0.05	n.s.
Whole body BMD g/cm^2^	0.93 (0.02)	1.06 (0.06)	0.96 (0.01)	0.98 (0.04)	<0.05	n.s.
Z-score	−1.67 (0.23)	−1.22 (0.28)	−1.42 (0.14)	−1.48 (0.18)	<0.05	n.s.
BMAD_LS g/cm^3^	0.20 (0.08)	0.24 (0.09)	0.21 (0.05)	0.24 (0.06)	<0.05	<0.05
Z-score	−1.39 (0.36)	−0.49 (0.40)	−1.29 (0.23)	−0.50 (0.25)	<0.05	<0.05
BMAD_hip g/cm^3^	0.28 (0.01)	0.31 (0.02)	0.27 (0.01)	0.27 (0.01)	<0.05	<0.05
Z-score	−0.88 (0.23)	−0.35 (0.37)	−1.36 (0.20)	−1.21 (0.24)	<0.05	<0.05
Transboys						
	Early-pubertal		Late-pubertal			
	0	36	0	36	*p*1	*p*2
aBMD_LS g/cm^2^	0.82 (0.04)	1.02 (0.07)	0.90 (0.02)	0.99 (0.02)	<0.05	<0.05
Z-score	−1.30 (0.43)	0.11 (0.58)	−0.68 (0.16)	−0.26 (0.22)	<0.05	<0.05
aBMD_hip g/cm^2^	0.83 (0.04)	1.02 (0.06)	0.88 (0.02)	0.96 (0.02)	<0.05	<0.05
Z-score	−0.82 (0.33)	0.59 (0.43)	−0.50 (0.12)	0.12 (0.16)	<0.05	<0.05
Whole body BMD g/cm^2^	0.94 (0.03)	1.11 (0.10)	1.02 (0.01)	1.10 (0.03)	n.s.	<0.05
Z-score	−1.06 (0.32)	0.21(0.43)	−0.30 (0.12)	−0.05 (0.16)	<0.05	<0.05
BMAD_LS g/cm^3^	0.22(0.01)	0.26 (0.01)	0.24 (0.01)	0.26 (0.01)	<0.05	<0.05
Z-score	−1.01 (0.49)	0.12 (0.51)	−0.61 (0.18)	−0.04 (0.18)	<0.05	<0.05
BMAD_hip g/cm^3^	0.28 (0.02)	0.32 (0.02)	0.30 (0.01)	0.32 (0.01)	<0.05	n.s.
Z-score	−0.71 (0.37)	0.01 (0.43)	−0.41 (0.14)	−0.10 (0.16)	<0.05	n.s.

aBMD and BMAD during 3 years of GnRHa plus gender-affirming hormone treatment. Values are presented as estimated marginal means ± standard error. *p1* represents the *P* value between start and after 3 years of treatment for the early-pubertal groups. *p2* represents the *P* value between start and after 3 years of treatment for the late-pubertal groups.

For changes per year of treatment see Fig. 2.

Abbreviations: aBMD, areal bone mineral density; BMAD, bone mineral apparent density; BMD, bone mineral density; LS, lumbar spine.

#### Bone mineral apparent density z-scores.

The BMAD z-scores of the lumbar spine significantly increased in all 4 groups (Fig. 3, Table 4). Z-scores of the femoral neck showed a significant increase in both groups of transgirls and in the early pubertal transboys. The increase of the z-score in late-pubertal transboys was not significant.

Three transgirls had a z-score of the femoral neck below −2 and 3 individuals had a z-score of the lumbar spine below −2 after 3 years of gender-affirming hormone treatment. None of the transboys had a z-score below −2 after 3 years of gender-affirming hormone treatment.

#### Serum bone markers.

**Figure 4.**
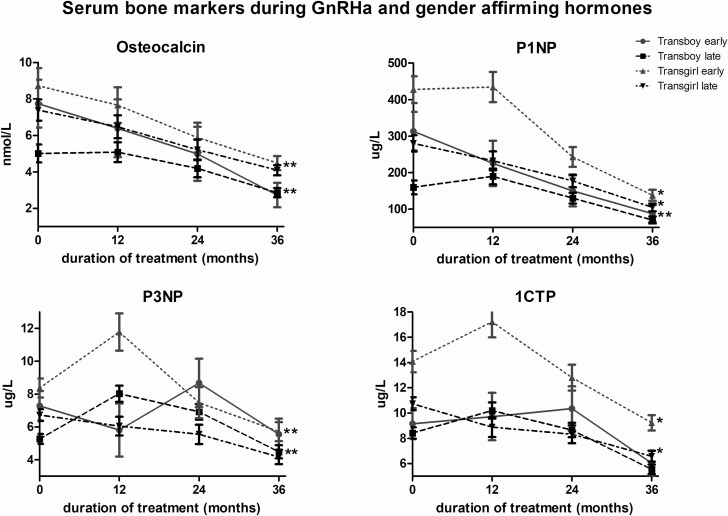
Estimated marginal means and standard error of the mean of osteocalcin, P1NP, P3NP, and 1CTP prior to and during 3 years of GnRHa + gender-affirming treatment in transgirls and transboys. Significant changes during the 3 years of GnRHa + gender-affirming treatment are indicated by an asteriks.

The mean serum levels of the bone markers prior to gender-affirming hormone administration are shown in Fig. 4. Serum levels of P1NP, P3NP, and 1CTP were significantly higher in the early pubertal transgirls than in the late-pubertal transgirls. In the transboys, baseline serum levels of P1NP and P3NP were significantly higher in the early pubertal group compared with the late-pubertal group. Levels of all 4 markers changed little in the late-pubertal transboys, whereas in the early pubertal transboys and late-pubertal transgirls, osteocalcin, P1NP, and P3NP showed a pronounced decrease during the first year of gender-affirming hormone treatment, after which levels stabilized. Remarkably, in the early-pubertal transgirls an initial increase in the P1NP, P3NP, and 1CTP levels was found followed by a decrease. After 3 years of gender-affirming hormone treatment, all 4 bone markers had significantly decreased in both early and late-pubertal transgirls. In transboys, osteocalcin, P1NP, and 1CTP significantly decreased. In both early and late-pubertal transboys, serum levels of P3NP did not significantly change.

## Discussion

This study examined the impact of puberty suppression and subsequent addition of gender-affirming hormones on bone development in adolescents diagnosed with gender dysphoria. At the start of GnRHa treatment, aBMD and BMAD values were within the normal range. However, transgirls had z-scores well below zero, whereas these were close to zero in transboys. This finding is consistent with previous studies (8, 10, 16-18) and may be explained by differences in lifestyle and exercise intensity between transgirls and transboys. A recent study showed that high-school transgirls have a higher intake of fast-food and are less physically active than transboys (19). In a different cohort of transgender adolescents we found vitamin D levels <50 nmol/L in 74% of transboys and 78% of transgirls starting GnRHa treatment ((9) and unpublished data). However, these findings do not explain why BMD z-scores are lower in transgirls than in transboys. Alternatively, it may be hypothesized that biological factors that act during intrauterine or early development and are involved in the development of gender dysphoria, are also related to bone development programming. For example, a whole-exome sequencing study in transgender individuals found 21 variants in 19 genes associated with estrogen activated pathways of sexually dimorphic brain development (20). These variants in estrogen receptor–activated pathways might also play a role in bone mineral acquisition.

During GnRHa treatment we observed a decline of aBMD and BMAD z-scores in line with previous studies (8-10). In transgirls a decrease of aBMD z-scores was also reported with the use of the anti-androgenic progestin cyproterone acetate (18). In contrast, 1 study showed that in transboys treated with the progestin lynestrenol for an average of 11.6 months aBMD z-scores were stable or increased (18). If these results are confirmed, also with more prolonged treatment duration, the better safety profile with regard to bone health is an important point to discuss with adolescents. In particular, older transboys who have already completed breast development may prefer lynestrenol to GnRHa treatment.

In most individuals with prolonged (3-4 years) GnRHa treatment, no further decrease in aBMD z-scores was observed in the last year, suggesting that z-scores might stabilize. Data from a larger cohort of adolescents treated with GnRHa for longer periods of time are needed, especially now that adolescents are presenting at younger ages at gender identity clinics and starting treatment at the onset of puberty.

During gender-affirming hormone treatment, a significant increase in the BMAD of the lumbar spine was found in all groups, and of the femoral neck in all but the late-pubertal transboys. In line with previous studies, BMAD z-scores were close to zero in transboys after 3 years of testosterone treatment (8-10). The increase in z-scores was most pronounced in the early pubertal transboys whose z-scores were slightly higher after 3 years of androgen treatment than at the start of GnRHa treatment.

The BMAD z-scores remained well below zero in transgirls in line with previous studies (8, 10). However, BMAD z-scores in early-pubertal transgirls increased more during estrogen treatment and were higher after 36 months than the scores reported by Vlot et al after 24 months (8). This might be due to the extra year of estrogen treatment in the current study, although the z-score of BMAD at the femoral neck no longer seemed to increase between 24 and 36 months. In contrast, the BMAD z-scores of the femoral neck in the late-pubertal transgirls were much lower after 36 months in the current study than previously reported (8). This may be due to the lower z-scores at the start of GnRHa treatment (−1.01 vs −0.44) and at the start of estrogen treatment (−1.36 vs −0.36) in the current study compared with the study by Vlot et al.

An important limitation of this study is the lack of an untreated control group. As discussed above, z-scores in transgirls were already well below 0 at the start of treatment, and these might have further decreased even without treatment, as low BMD was also observed in adult transwomen before the start of any treatment (16, 17).

Another issue is which reference population should be used to calculate BMD or BMAD z-scores. In transgirls who started treatment in early puberty, bone architecture may be more similar to that of cisgender females than to cisgender males. A recent study did not find changes in cortical bone geometry in response to estrogen treatment in adult transwomen, but the authors suggested that this might have been different if they had started treatment during puberty (21).

GnRHa are not only used in transgender children, but also in other populations, mainly in children with precocious or early puberty. A recent publication from an international consortium on the use of GnRHa concluded from the available evidence in this group that the treatment was safe with regard to bone mineral density, with attenuated bone mineral accrual reported during treatment but recovery by late adolescence (22). Different findings in children with precocious puberty compared with transgender adolescents may be due to the different timing of GnRHa treatment, the use of gender-affirming hormones, with current estradiol dose possibly insufficient (23), versus endogenous puberty, and due to differences in baseline BMD between the groups.

In transgirls and early-pubertal transboys, all bone markers decreased during the first year of GnRHa treatment while BMD levels remained stable. However, in the late-pubertal transboys bone turnover markers were lower at baseline and did not change. This suggests that the decline of the bone markers during GnRHa treatment may not be due to reduced bone mineral accrual but may rather reflect reduced growth velocity after initiating treatment. The late-pubertal transboys had likely already reached (near) adult height, which could explain the lower and stable levels of bone turnover markers. We previously observed a similar decrease of alkaline phosphatase during GnRHa treatment, but only in those who had not yet completed growth (24). The opposite effect was seen during the first year of treatment with gender-affirming hormones, where bone markers increased in the early pubertal transgirls, who likely had most growth potential. In adults, changes in P1NP were also found to be only weakly correlated to changes in BMD in transwomen and not significantly correlated in transmen (25). A previous study of bone turnover markers in adolescents observed a similar pattern of changes in P1NP and 1CTP to the current study (8). However, changes in osteocalcin were only seen in late-pubertal transboys, possibly due to the small number of subjects in that study with large interindividual differences in the changes of osteocalcin levels (8).

Based on the current study we propose that it is sufficient to perform DXA scans at the start of GnRHa treatment, every 2 years during GnRHa treatment, at the start of gender-affirming hormone treatment, and then every 2 to 3 years. Adolescents should be counseled on the importance of weight-bearing exercise, an adequate dietary calcium intake, sufficient sunlight exposure to ensure adequate vitamin D levels, or vitamin D supplementation (26). In addition, it is important to ensure an adequate estrogen dose resulting in physiological serum estradiol levels. Routine measurement of bone turnover markers does not seem to be useful for monitoring bone health.

In conclusion, treatment with GnRHa results in a stabilization and maintenance of previously achieved bone mass in the lumbar spine but a small decrease in BMAD of the femoral neck of the nondominant hip. Gender-affirming hormone treatment increases bone accretion and normalizes the age- and sex-specific BMAD z-scores in transboys. Transgirls had lower BMAD z-scores, especially the late-pubertal group, but as z-scores were already lower at baseline, this may be due to other factors than the endocrine treatment, such as lifestyle factors. The consequences of lower BMD for long-term bone health in these individuals remains unclear. Future studies should evaluate peak bone mass in those who started treatment as adolescents and investigate clinically important outcomes such as fracture risk in this population.

## Data Availability

The datasets generated during and/or analyzed during the current study are not publicly available but are available from the corresponding author on reasonable request.

## Abbreviations

1CTP: carboxyterminal cross-linked telopeptide of type I collagen
aBMD: areal bone mineral density
ANOVA: analysis of variance
BMAD: bone mineral apparent density
BMD: bone mineral density
CV: coefficient of variation
DXA: dual-energy x-ray absorptiometry
GnRH: gonadotropin-releasing hormone
GnRHa: gonadotropin-releasing hormone analogue
P1NP: N-terminal propeptide of type-1 collagen
PBM: peak bone mass

## Additional Information

***Disclosure Summary:*** The authors have nothing to disclose.
